# γδ T cell activation may contribute to antitumor immunity stimulated by intralesional BCG immune therapy for cutaneous melanoma metastases

**DOI:** 10.1186/2051-1426-3-S2-P312

**Published:** 2015-11-04

**Authors:** Romela Ramos, Agnes Lee, Alfred Chan, Keith S Bahjat, Leland Foshag, Mark Faries, Delphine Lee, Peter Sieling

**Affiliations:** 1 John Wayne Cancer Institute, Santa Monica, CA, USA; 2 Earle A. Chiles Research Institute, Robert W. Franz Cancer Research Center, Providence Cancer Center, Portland, OR, USA

## 

In-transit melanoma metastasis can lead to significant locoregional toxicity, due to painful, bleeding, or necrotic lesions, which may become superinfected. Intralesional immunotherapy is a rational approach to such disease as the lesions are accessible and provide a source of ideally matched tumor antigen to facilitate local, and possibly systemic, immune responses. *Mycobacterium bovis* Bacille Calmette-Guerin (BCG) is one of several local therapy options recommended in the NCCN Guidelines, version 3.2015 for stage III melanoma. Direct injection of BCG into metastatic melanoma lesions in the skin (intralesional BCG or ILBCG) resulted in 90% regression of injected lesions and 17% regression of uninjected lesions in immunocompetent patients. Although the mechanism through which BCG functions to eliminate tumor is unclear, it is likely immune mediated and involves T cells. BCG and other mycobacteria have a propensity to stimulate T cells expressing Vγ9Vδ2 T cell receptors, thus, we hypothesized that γδ T cells contribute to BCG-mediated antitumor immunity in in-transit melanoma. We detected an increase in Vγ9 T cell infiltration in uninjected ILBCG lesion of one patient during the course of ILBCG therapy as well as increased gene expression of BTN3A1 (butyrophilin), a protein that facilitates γδ T cell activation, suggesting the prevalence and migration of γδ T cells into skin lesions. We further found that butyrophilin, and γδ TCR expression were upregulated in BCG-stimulated PBMC of melanoma patients (n=5). BCG also induced proliferation and activation of γδ T cells as evidenced by a 2,000-fold increase in Vγ9 T cell proliferation and a 9- and 19-fold increase in CD69 and HLA-DR expression in Vγ9 T cells, respectively. Moreover, BCG stimulated a 5 to 21-fold increase in IFN-γ producing γδ T cells in comparison to unstimulated PBMC (n=3), whereas 0 to 2.2-fold increases in IFN-g producing αβ T cells were detected in response to BCG. Vγ9 T cells expanded with BCG made more granzyme A (p < 0.04) and granzyme B (p < 0.0008) compared to unstimulated PBMC. Finally, an in-transit melanoma tumor that was regressing after ILBCG yielded a 117-fold increase of γδ T cells producing IFN-γ relative to a non-regressing tumor from the same patient. These data suggest that immune therapy approaches that promote tumor recognition by γδ T cells and boost effector functions are feasible and that further studies should be performed to define the role of γδ T cells BCG-mediated regression for in-transit melanoma patients.

